# Transcriptome analysis of the oil-rich seed of the bioenergy crop *Jatropha curcas *L

**DOI:** 10.1186/1471-2164-11-462

**Published:** 2010-08-06

**Authors:** Gustavo GL Costa, Kiara C Cardoso, Luís EV Del Bem, Aline C Lima, Muciana AS Cunha, Luciana de Campos-Leite, Renato Vicentini, Fábio Papes, Raquel C Moreira, José A Yunes, Francisco AP Campos, Márcio J Da Silva

**Affiliations:** 1 Centre for Molecular Biology and Genetic Engineering, State University of Campinas, UNICAMP, Campinas, SP, Brazil; 2 Biochemistry and Molecular Biology Department, Federal University of Ceará, UFC, Fortaleza, CE, Brazil; 3 Departament of Genetics, Evolution and Bioagents, State University of Campinas, UNICAMP, Campinas, SP, Brazil; 4 Research and Development Center, CENPES-PETROBRAS, Rio de Janeiro, RJ, Brazil

## Abstract

**Background:**

To date, oil-rich plants are the main source of biodiesel products. Because concerns have been voiced about the impact of oil-crop cultivation on the price of food commodities, the interest in oil plants not used for food production and amenable to cultivation on non-agricultural land has soared. As a non-food, drought-resistant and oil-rich crop, *Jatropha curcas *L. fulfils many of the requirements for biofuel production.

**Results:**

We have generated 13,249 expressed sequence tags (ESTs) from developing and germinating *Jatropha *seeds. This strategy allowed us to detect most known genes related to lipid synthesis and degradation. We have also identified ESTs coding for proteins that may be involved in the toxicity of *Jatropha *seeds. Another unexpected finding is the high number of ESTs containing transposable element-related sequences in the developing seed library (800) when contrasted with those found in the germinating seed library (80).

**Conclusions:**

The sequences generated in this work represent a considerable increase in the number of sequences deposited in public databases. These results can be used to produce genetically improved varieties of *Jatropha *with increased oil yields, different oil compositions and better agronomic characteristics.

## Background

The need to reduce greenhouse gas emissions and provide fuel security has increased the demand for oil-rich plants as raw materials for biodiesel production. Although vegetable oils have long been used for food, the ideal crop source for biodiesel products should consider other ecological, environmental and ethical concerns. Ideally, the entire process, from cultivation to fuel burning in engines, should favour carbon sequestration, reduce water needs and promote energy efficiency. Moreover, the impact of oil crops for biodiesel production on the prices of food commodities is a matter of concern. Ideally, such crops should be non-edible and grown on non-agricultural lands so that they do not compete for soil with food crops and do not affect the price of food commodities.

*Jatropha curcas *L. (family Euphorbiaceae) is a perennial, drought-resistant and non-food oilseed crop that has high oil content and fulfils many of the requirements for biodiesel production. *Jatropha *is currently one the most promoted oilseed crops and its seeds have an oil content of up to 50% [1]. Its major fatty acids are oleic acid (34.3-45.8%; 18:1), linoleic acid (29.0-44.2%; 18:2), palmitic acid (14.1-15.3%; 16:0) and stearic acid (3.7-9.8%; 18:0) [2]. Because *Jatropha *seeds accumulate very high levels of protein in the endosperm, the residue obtained after oil extraction may potentially be used for animal feed, adding extra value to the crop.

Despite the recent attention that *Jatropha *has received as an oil source for biodiesel products, its potential has not yet been fully realised. Unlike other oil crops such as soybean, maize, rapeseed and sunflower, there are no agronomically improved varieties of *Jatropha *[3]. Potential areas of improvement are increased oil yield and reduced seed toxicity. Genomic and transcriptomic resources have been generated to accelerate the genetic improvement of many crops [4]. Although a privately held company announced the completion of the *J. curcas *genome, the data have not been made publicly available, and transcript resources in public databases are scarce. To bridge this gap, we have sampled the transcriptome of developing and germinating *Jatropha *seeds to unveil the gene repertoires of *J. curcas *related to the following: (1) oil accumulation during seed development and oil breakdown during germination; and (2) proteins possessing toxic, anti-nutritional or allergenic properties and enzymes involved in the biosynthetic pathway for phorbol esters, the major toxic components of *Jatropha *seeds.

Here, we have sequenced 13,249 ESTs from two cDNA libraries of *J. curcas *developing (JD) and germinating (JG) seeds. Sequencing of transcripts from these two contrasting developmental phases has allowed us to assess differential expression and discover most genes that are related to lipid metabolism. We have used these sequences to reconstruct the main metabolic pathways related to lipid synthesis and breakdown in *J. curcas*.

The sequences presented in this work represent a considerable increase in the total number of *J. curcas *ESTs deposited in GenBank. These results will be useful for further biotechnological interventions related to *Jatropha *seeds.

## Results and Discussion

### *Jatropha seed *EST database

We have generated cDNA libraries from pools of developing (19, 26, 33 and 40 days after pollination - DAP) and germinating endosperm (24, 36, 48 and 72 hours after imbibition - HAI) of *Jatropha curcas *seeds. We have sequenced 7,320 ESTs from the developing pool (JD) and 5,929 from the germinating pool (JG), totalling 13,249 high-quality ESTs. The lengths of the ESTs after trimming ranged from 100 to 848 bp, with an average size of 561.5 bp. The ESTs from both libraries were assembled together into 1,606 contigs and 5,677 singletons, resulting in 7,283 unisequences.

**Figure 1:**
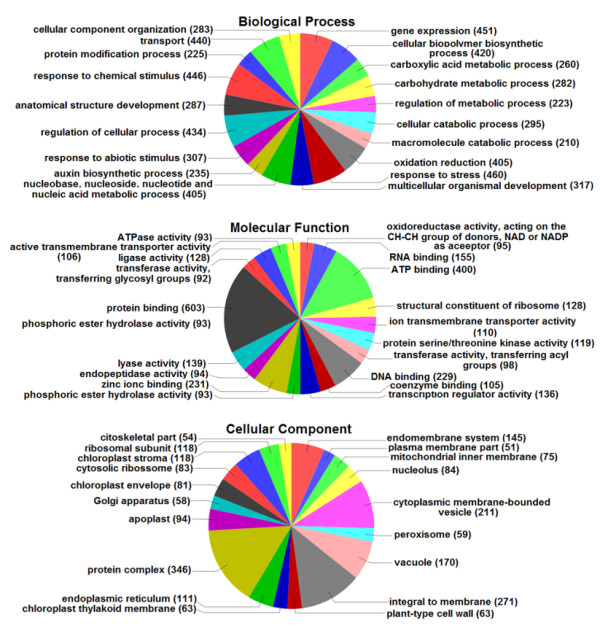
**Functional Classification**. Functional classification of *Jatropha *unisequences obtained from developing and germinating seed libraries.

All unisequences were aligned against the non-redundant (NR) protein database of GenBank using BLASTX with an e-value cut-off of 1e-10. We found matches for 4,928 unisequences (67.7%). The remaining 2,363 unisequences with no matches in the NR database were subjected to gene prediction analysis using ESTScan. This approach resulted in ORF predictions for 1,766 unisequences. The combination of the NR matches with the ESTScan predictions resulted in 6,694 (91.9%) putative protein-coding unisequences, of which 161 contain a complete ORF (full-length sequences). Blast2GO [5] categorization was performed, as shown in Figure 1 (See additional file 1 for automatic annotation of all unisequences using Blast2GO). It is noteworthy to highlight the oxidoreductase activity and auxin biosynthesis in this annotation, both of which are key processes in early plantlet establishment related to energy uptake from seed reserves and growth, respectively. Important signal transduction elements were categorized such as putative serine/threonine protein kinases (119 unisequences) and transcription factors (89 unisequences). Several predicted cellular components have been identified as membrane-associated proteins.

**Figure 2:**
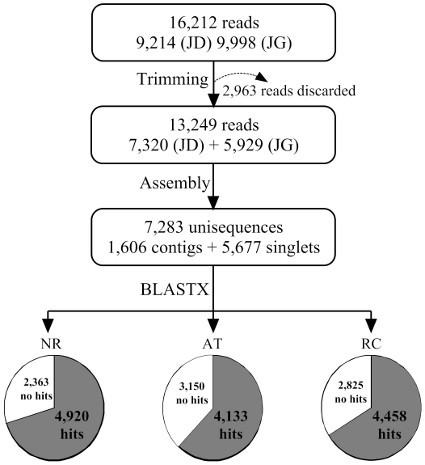
**Bioinformatics pipeline for EST clustering, assembly and annotation**. We generated 16,112 reads (9,214 from JD and 6,998 from JG). All reads were trimmed and 13,249 reads were kept for clustering. The clustering resulted in 7,283 valid clusters that were aligned against the GenBank non-redundant protein database (NR), the *Arabidopsis thaliana *predicted proteome (At) and the *Ricinus communis *predicted proteome (Rc).

The detailed bioinformatic protocol is shown in Figure 2. All ESTs were deposited in the dbEST division of GenBank under accession numbers GT969394 to GT982642 (See additional file 2 for the correspondence between internal unisequence IDs and GenBank accession numbers).

### Categories of the most abundantly expressed ESTs in developing and germinating *Jatropha seeds*

Because our cDNA libraries are not normalised, we were able to use the EST abundance in the resulting contigs to estimate differential expression levels of transcripts in each cDNA pool. Among the top 20 most highly expressed transcripts in JD, there are three different transcripts coding for storage proteins belonging to the 11 S globulin family (Table 1; Contig153, Contig81 and Contig818) and one transcript coding for an aspartyl protease (Table 1; Contig254), which is known to be involved in processing the precursors of storage proteins into mature proteins [6].

**Table 1:** The 20 most highly expressed transcripts in the developing endosperm

Unisequence	Gene product	JD	JG	p-value
Contig452	Tubulin alpha-2 chain	68	31	6.899e-03
Contig153	11 S globulin seed storage protein 2	56	0	4.109e-15
Contig667	Putative chloroplast transcript	56	9	1.345e-07
Contig100	Ribosome protein 1	52	17	6.409e-04
Contig206	Legumin A	47	0	8.566e-13
Contig826	Polyubiquitin	44	113	5.635e-12
Contig254	Aspartyl protease family protein	42	0	1.664e-11
Contig268	Chloroplast ribosomal protein L2	35	5	1.696e-05
Contig851	Large subunit of RUBISCO	33	0	3.469e-09
Contig1	Unknown	32	0	6.278e-09
Contig81	Legumin B/11 S globulin precursor	27	0	1.220e-07
Contig284	Myb family transcription factor	26	0	2.207e-07
Contig367	ATP synthase gamma chain, mitochondrial	26	12	1.075e-01
Contig461	Polygalacturonase (pectinase)	22	3	6.460e-04
Contig571	Retrotransposon protein	22	1	2.675e-05
Contig818	11 S globulin seed storage protein 2	22	0	2.369e-06
Contig202	Adenosylmethionine decarboxylase	21	1	4.650e-05
Contig1382	Aquaporin	21	112	5.523e-21
Contig203	Malate dehydrogenase, cytosolic	20	5	1.238e-02
Contig596	Chloroplast sequence	20	1	8.070e-05

Annotation and expression levels of the 20 most highly expressed transcripts in the developing endosperm (JD). The columns JD and JG shows the number of ESTs for each unisequence in developing and germinating endosperm, respectively. The p-value refers to the Audic-Claverie statistics for differential expression

These findings are consistent with the high levels of storage proteins in the endosperm of *J. curcas *seeds [7]. Reflecting the intense catabolic activity within germinating seeds related to the mobilisation of protein reserves [8], three of the most abundant transcripts code for proteinases of the cysteine proteinase family (Table 2; Contig1184, Contig515 and Contig1058), which are known to be involved in protein mobilisation during seed germination [9]. Another abundant transcript codes for a cysteine proteinase inhibitor (Table 2; Contig724), which is thought to be involved in spatial and temporal control of storage protein mobilisation by cysteine proteinases during seed germination [10]. Transcripts related to breakdown of the oil and carbohydrate reserves, such as acetyl-CoA C-acyltransferase, are also represented amongst the most abundant transcripts in the germinating library (Table 2; Contig1038).

**Table 2:** The 20 most highly expressed transcripts in the germinating endosperm

Unisequence	Gene product	JG	JD	p-value
Contig1378	Subunit 8 of the mitochondrial F(O) ATP synthase complex.	129	0	8.0e-46
Contig826	Polyubiquitin	113	44	5.6e-12
Contig1113	NADP-dependent oxidoreductase, putative	112	0	6.9e-40
Contig1382	Tonoplast intrinsic protein (TIP) gamma	112	21	5.5e-21
Contig1362	Allene oxide synthase	74	0	1.3e-26
Contig1446	Isocitrate lyase	44	0	3.9e-16
Contig1038	Acetyl-CoA C-acyltransferase	42	5	1.9e-10
Contig1516	60 S ribosomal protein L13A	41	14	8.3e-06
Contig1092	Epoxide hydrolase	39	3	4.8e-11
Contig724	Cysteine protease inhibitor, putative	33	2	5.4e-10
Contig452	Tubulin alpha-2 chain	31	68	6.9e-03
Contig587	Ethylene-responsive transcription factor RAP2-12	29	11	4.3e-04
Contig1194	Cysteine-type peptidase	29	0	6.7e-11
Contig1392	Fructose-bisphosphate aldolase	29	0	6.7e-11
Contig40	L-asparagine amidohydrolase, putative	28	4	6.4e-07
Contig1606	Catalase	28	1	2.5e-09
Contig515	Cysteine proteinase, putative	25	1	2.6e-08
Contig1058	Cysteine-type peptidase	25	1	2.6e-08
Contig1108	26 S ribosomal RNA gene	25	2	2.0e-07
Contig1259	Cellular repressor of E1A-stimulated genes (CREG) family	25	0	1.7e-09

Annotation and expression levels of the 20 most highly expressed transcripts in the germinating endosperm (JG). The columns JG and JD shows the number of ESTs for each unisequence in germinating and developing endosperm, respectively. The p-value refers to the Audic-Claverie statistics for differential expression

### Transposable elements are highly active in developing *Jatropha seeds*

The annotation revealed surprising differences between the number of transposable element (TE) sequences in the JD and JG libraries, both in terms of the number of unisequences containing a TE-related sequence (475 unisequences in JD and 42 unisequences in JG) and in terms of their relative expression levels (800 ESTs in JD and 80 ESTs in JG). Suppression of transposon silencing in the endosperm during seed development has been described in *Arabidopsis *[11]. This suppression was concluded to result from genome-wide demethylation of maternal alleles in the endosperm. *Arabidopsis *uses this mechanism to imprint expression of maternal alleles in the endosperm. In that work, the authors suggest that temporally regulated transposon activation carries a moderately low cost to *Arabidopsis *because it has few functional transposons and the endosperm genome is not transmitted to the next generation.

Unlike *Arabidopsis*, *Jatropha *has many active transposons, as evidenced in our JD library (800 ESTs). Further investigation will be necessary to verify if the high number of TE elements in JD is related to demethylation of the maternal alleles during development.

### ESTs related to toxic components

The seeds of *J. curcas *are extremely toxic to a wide range of animals, but the biochemical basis for this toxicity is still under investigation [3]. Curcin, a class I ribosome-inactivating protein (RIP), is present in *J. curcas *seeds but its toxicity is at least 1000-fold lower than that of ricin, a class II RIP from *Ricinus communis *[12]. In our libraries we were unable to find transcripts coding for curcin homologues, but a preliminary proteomic analysis of developing and germinating seeds led us to identify five curcin isoforms (data not shown). Several transcripts coding for protein inhibitors of serine (SEI, Table 3) and cysteine (CPI, Table 3) proteinases were found in both libraries. Although the presence of these protein inhibitors may raise biosafety concerns regarding use in animal feed, it is unlikely that they play a major role in toxicity because their effects are relatively mild (King et al., 2009).

**Table 3:** Enzymes related to toxicity in seeds

Symbol	Enzyme	Unisequences	JD	JG	p-value	NJD	NJG
SEI	Serine-type inhibitor	GJCCJC2052B11.b	1	0	6.1e-01	1.36	0
	endopeptidase						
CPI	Cysteine protease	Contig724	2	33	5.4e-10	2.73	55.65
	inhibitor	GJCCJC2009G08.b	1	0	6.1e-01	1.36	0
		GJCCJC2052B11.b	1	0	6.1e-01	1.36	0
		JGCCJG2001C08.b	0	1	4.0e-01	0	1.68
		JGCCJG2009F10.b	0	1	4.0e-01	0	1.68
		JGCCJG2022B04.b	0	1	4.0e-01	0	1.68
		JGCCJG2042G12.b1	0	1	4.0e-01	0	1.68
		JGCCJG2063F11.b	0	1	4.0e-01	0	1.68
		JGCCJG2067E09.b	0	1	4.0e-01	0	1.68
FPS2	Farnesyl diphosphate	GJCCJC2080E09.b	1	0	6.1e-01	1.36	0
	synthase 2						
GGR	Geranylgeranyl-	Contig785	1	1	8.4e-01	1.36	1.68
	diphosphate synthase	GJCCJC2040E06.b	1	0	6.1e-01	1.36	0

Uniquences assigned to enzymes related to toxicity in seeds. The columns JD and JG shows the number of ESTs for each unisequence in developing and germinating endosperm, respectively. The p-value refers to the Audic-Claverie statistics for differential expression. NJD shows the number of ESTs in JD normalised to 10,000 reads. NJG shows the number of ESTs in JG normalised to 10,000 reads.

The seeds of several Euphorbiaceae are known to be a rich source of powerful allergens of the 2 S albumin family, rendering the cultivation, handling and consumption of seeds a serious health hazard. Transcripts coding for 2 S albumins were found only in the JD library, which is consistent with the role of these methionine- and cysteine-rich storage proteins as a specialised reserve of sulphur for the growing seedling [13]. Although the issue of allergenicity in connection with *J. curcas *seeds has not yet been raised, the presence of 2 S albumins in seeds may become an important health issue given the interest in the use of seedcake, which is the byproduct remaining after seeds are used as biodiesel source, as animal feed. Therefore, the allergenicity of protein fractions from the seeds and other tissues merits investigation.

Another important toxic component of the *J. curcas *seeds is a group of diterpene esters termed phorbol esters, which have structures based on a tetracyclic carbon skeleton known as tigliane (Haas *et al*., 2002). Phorbol esters are thought to be the major toxic components of seeds. These compounds mimic the action of diacylglycerol (DAG), an activator of protein kinase C, which in turn regulates different signal transduction pathways and other cellular metabolic activities and thereby amplifying the efficacy of carcinogens (Goel *et al*., 2007). We have searched our libraries for transcripts that could increase our understanding of the phorbol ester biosynthesis pathway, and found several transcripts coding for enzymes involved in synthesis of the major subclasses of terpenoids. The terpenoids are synthesised from the basic five-carbon unit isopentenyl diphosphate (IPP) and the initial prenyl (allylic) diphosphate, dimethylallyl diphosphate (DMAPP), which is formed by the isomerisation of IPP. Prenyltransferases catalyse alkylation of one or more molecules of IPP (C5) with DMAPP (C5) to produce geranyl diphosphate (GPP; C10), farnesyl diphosphate (FPP; C15) and geranyl geranyl diphosphate (GGPP; C20). Transcripts coding for farnesyl-diphosphate synthase (FPS2, Table 3) and geranylgeranyl-diphosphate synthase (GGR, Table 3) were found in our libraries.

The identification of genes related to *Jatropha *toxic components can accelerate the development of genetic strategies to produce varieties of *J. curcas *with low toxicity, increasing the possibility of using the seed as animal feed and obtaining a plant with improved agricultural handling characteristics.

### Reconstruction of metabolic pathways related to oil accumulation in seeds

We have used KAAS [14] and KOBAS [15] to annotate automatically *Jatropha *ESTs coding for orthologues to plant enzymes in the fatty acid biosynthesis, fatty acid degradation, triacylglycerol biosynthesis and triacylglycerol degradation pathways. ESTs related to plant lipid metabolism were also manually annotated using PlantCYC [16] and the Arabidopsis Lipid Database [17] (TBLASTN/BLASTX bidirectional best hit; e-value 1e-10). We have integrated and compiled these data to propose schematic metabolic pathways that lead to oil accumulation in *Jatropha *seeds.

**Figure 3:**
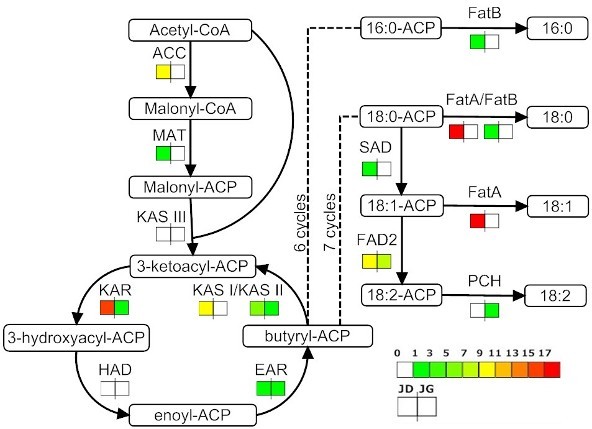
**Fatty acid biosynthesis pathway**. *Jatropha *enzymes found in the fatty acid biosynthesis pathway. The icons beside each enzyme name show the total number of *Jatropha *ESTs corresponding to that enzyme in JD and JG, separately. KAS I participates from the 2nd to the 6th carbon-chain extension cycles. KAS II participates in the 7th carbon-chain extension cycle. Both FatA and FatB can catalyse conversion of 18:0-ACP into stearic acid. The fatty acids produced by this pathway are 16:0 (palmitic acid), 18:0 (stearic acid), 18:1 (oleic acid) and 18:2 (linoleic acid). See additional file 4 for the unisequences assigned to each enzyme.

While most ESTs coding for fatty acid biosynthesis enzymes were found in JD, the fatty acid degradation pathway is enriched in JG ESTs, which is expected considering that these stages are dedicated to oil accumulation and breakdown, respectively. In the fatty acid biosynthesis pathway, we found a considerable number of ESTs coding for almost all enzymes (except KAS III and HAD, Figure 3). More specifically, we found ESTs coding for enzymes catalysing reactions that ultimately produce oleic and stearic (FatA, Table 4), linoleic (PCH, Table 4) and palmitic (FatA and FatB, Table 4) acids, the main constituents of *Jatropha *seed oil. Additionally, we found 12 ESTs coding for oleoyl-ACP desaturase (FAD2, Table 4), which catalyses the polyunsaturation of oleoyl-ACP (18:1) to linoleoyl-ACP (18:2). Because oleic and linoleic acids are the major constituents of *Jatropha *oil, this enzyme is a potential biotechnological target for modulation of *Jatropha *oil composition.

**Table 4:** Enzymes related to oil accumulation and breakdown in seeds

Symbol	Enzyme	Unisequences	NJD	NJG
**Fatty Acid Biosynthesis**				

FatA	Acyl-ACP thioesterase A	2	25.95	0
FatB	Acyl-ACP thioesterase B	1	1.36	0
ACC	Acetyl-CoA carboxylase	4	10.91	0
EAR	Enoyl-ACP reductase	2	2.72	1.68
HAD	Hydroxyacyl-ACP dehydrase	0	0	0
KAR	Ketoacyl-ACP reductase	5	16.36	1.68
KAS I	Ketoacyl-ACP synthase I	4	9.54	0
KAS II	Ketoacyl-ACP synthase II	2	5.45	1.68
KAS III	Ketoacyl-ACP synthase III	0	0	0
MAT	Malonyl-CoA ACP transacyclase	1	1.36	0
FAD2	Oleoyl-ACP desaturase	2	9.55	8.43
PCH	Palmitoyl-CoA hydrolase	1	0	1.68
SAD	Stearoyl-ACP desaturase	1	1.36	0

**Triacylglycerol Biosynthesis**				

LAT	1-Acylglycerol-3-phosphate-O-acyltransferase	1	1.36	0
DGAT	Acyl-CoA:diacylglycerol acyltransferase	2	1.36	1.68
GPAT	Glycerol-3-phosphate acyltransferase	1	1.36	0
PDAT	Phospholipid:diacylglycerol acyltransferase	1	0	1.68
PP	Phosphotidate phosphatase	0	0	0
OLE	Oleosin	2	4.09	0

**Triacylglycerol Degradation**				

ML	Monoacylglycerol lipase	1	1.36	0
PLAS	Peroxysomal long-chain acyl-CoA synthetase	5	2.72	21.86
PFAT	Peroxisomal fatty acid/acyl-CoA transporter	0	0	0
TL	Triacylglycerol lipase	14	21.82	40.45

**Fatty Acid Degradation**				

FADA	Acetyl-CoA acyltransferase	6	8.19	106.14
ATOB	Acetyl-CoA C-acetyltransferase	2	1.36	1.68
ADH	Alcohol dehydrogenase	1	2.72	1.68
ACADM	Acyl-CoA dehydrogenase	2	0	6.74
ACOX	Acyl-CoA oxidase	4	4.09	10.1
ALDH3A2	Aldehyde dehydrogenase (NAD+)	4	8.19	15.17
DCR	Dienoyl-CoA reductase	5	2.72	18.46
PAAG	Enoyl-CoA hydratase	12	9.53	47.19
ACSL	Long-chain acyl-CoA synthetase	4	2.72	3.36

Number of unisequences assigned to enzymes of metabolic pathways related to oil accumulation and breakdown in the seeds. NJD shows the number of ESTs in JD normalised to 10,000 reads. NJG shows the number of ESTs in JG normalised to 10,000 reads. See additional file 4 for the unisequence names and AC statistics.

Another important lipid class is the acylglycerols, which act as an energy reserve in many organisms and are the major components of seed storage oils. The most common acylglycerol in seed oils is triacylglycerol (TAG). Upon arrival in the cytoplasm, free FAs become esterified to coenzyme A (CoA) and serve as substrates for TAG synthesis from *sn*-glycerol-3-phosphate (see additional file 3: TAG_biosynthesis.jpg). After synthesis of 1,2-DAG, the formation of TAG can occur in two ways. In one pathway, diacylglycerol acyltransferase (DGAT, Table 4) transfers an acyl group from acyl-CoA to *sn*-3 of DAG to form TAG. We found one EST in JD and one EST in JG coding for DGAT. Another pathway involves a phospholipid:diacylglycerol acyltransferase (PDAT, Table 4) that utilises phospholipid as the acyl donor in TAG formation. The contribution of PDAT to the formation of TAGs in *Jatropha *oil is unknown, but its role in the castor bean has been identified as preferential incorporation of Δ-12-modified fatty acids.

After biosynthesis, pools of TAG can be stored in the mature seed in the form of oil bodies surrounded by a single monolayer membrane that is most likely generated through budding of the outer ER membrane. The membrane contains proteins known as oleosins, which are thought to stabilise the oil body during desiccation of the seed [18]. We found three ESTs coding for oleosins (OLE, Table 4) similar to the *Arabidopsis *oleosin AT4G25140.1.

### Reconstruction of metabolic pathways related to oil breakdown in seeds

**Figure 4:**
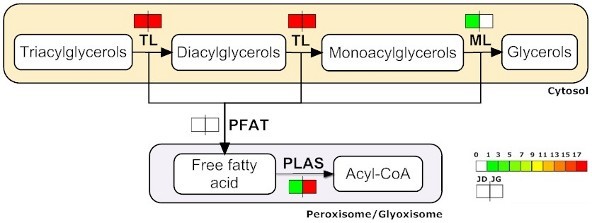
**Triacylglycerol degradation pathway**. *Jatropha *enzymes found in the triacylglycerol degradation pathway. The icons beside each enzyme name show the total number of *Jatropha *ESTs corresponding to that enzyme in the JD and JG, separately. See additional file 4 for the unisequences assigned to each enzyme.

We have used the same method described above to propose pathways related to oil breakdown. During germination, the embryo uses TAGs accumulated during development as an energy source. To this end, TAGs must be metabolised to free fatty acids (Figure 4) and then to acetyl-CoA.

Specialised lipases named TAG lipases appear to be induced during germination in most species [18]. The function of these lipases is to hydrolyse TAG into fatty acids and the intermediate product: diacylglycerol or monoacylglycerol [19]. As expected, we identified a large number of triacylglycerol lipases in our JG library (TL, Table 4).

Free fatty acids are metabolised into acetyl-CoA in the endoplasmic reticulum. We have found ESTs coding for all enzymes in the fatty acid degradation pathway. We also found a large number of ESTs for an acetyl-CoA C-acyltransferase, which catalyses acyltransferases reactions (FADA, Table 4).

## Conclusions

Here we have sequenced and annotated a collection of ESTs from libraries of developing and germinating seeds of *Jatropha curcas*. We identified ESTs related to transposable elements, toxic components and oil accumulation and breakdown in *Jatropha *seeds. *Jatropha curcas *is a species with agricultural relevance due to its oil-rich seeds, which can be used for biodiesel fuel production to help the world meet its energy needs in times of shrinking fossil fuel deposits. Given this economic significance, *Jatropha *plants require agronomic improvement. To achieve this goal, genomic information pertaining to the genes expressed in developing and germinating seeds, such as that described in this paper, is likely to be used. The need for such information is especially true for genes that are putatively involved in oil synthesis, accumulation and breakdown and those related to seed toxicity. For example, *Jatropha *plants can be genetically engineered to produce transgenic plants with improved oil content and/or composition. Methods can be applied to engineer the entire oil synthesis pathway, to increase expression of enzymes responsible for the synthesis of the sought-after fatty acids--oleic and linoleic acids--and to decrease expression of enzymes responsible for the breakdown of such energy-rich compounds. Moreover, *Jatropha *plants can be genetically improved using traditional or modern plant improvement methods to create varieties with reduced expression of potentially toxic compounds such as curcins, 2 S albumins and the enzymes involved in phorbol ester biosynthesis.

## Methods

### Biological material

*Jatropha curcas L*. developing fruits were harvested at 19, 26, 33 and 40 days after pollination. Fruits were dissected and the seeds were decoated to collect the endosperm, comprising developmental stages I to IV. *Jatropha *quiescent seeds were washed with 70% ethanol, decoated and sterilised with 3% sodium hypochlorite solution and 0.001% Tween-20 for five minutes. After five washes in sterile deionised water, *Jatropha *seeds were imbibed and germination was carried out in a 15-cm Petri dish on moistened filter paper at 28°C under constant darkness. We collected the endosperm at 24, 36, 48 and 72 hours after imbibitions (HAI), comprising germination stages I to IV, respectively.

### RNA isolation and library construction

Total RNA was extracted using the CONCERT Plant RNA Purification Reagent (Invitrogen) from the developing endosperms at 19, 26, 33 and 40 days after pollination (DAP) and from the germinating endosperms at 24, 36, 48 and 72 hours after imbibition (HAI). RNA samples from the developing and germinating endosperms were mixed in an equimolar concentration into two pools, respectively. The two cDNA libraries were constructed using the CloneMiner cDNA Library kit (Invitrogen) following the manufacturer's instructions.

### Sequencing

The EST library was sequenced using the BigDye terminator v 3.1 kit and an automated DNA capillary sequencer (ABI PRISM 3700 DNA Analyzer - Applied Biosystems). All ESTs were 5'-sequenced using the M13F primer (5'-TGTAAAACGACGGCCAGT-3').

### Cleaning and assembly of *Jatropha curcas *L. ESTs

We used the Phred base caller software to extract sequence and quality files from chromatograms. Next we used the Baudet *et al. *[20] EST cleaning pipeline to pre-process ESTs and prepare them for assembly. This pipeline accounts for plasmid similarity, polyA/polyT regions, low base quality and slippage. After identifying the positions of all of those features, it extracts the largest clean region of each EST. Sequences lacking a 100 bp clean region were discarded. We then used CAP3 [21] to cluster and assemble the clean sequences into contigs and singlets (unigenes). For this step, we set the parameters to require an identity of at least 95% over 50 bp to detect pairwise similarities.

### Unisequence annotation

After clustering and assembly, we used BLAST to search for similarities between our unigenes and sequences deposited in public databases. We used the predicted complete proteome of the model eudicot *Arabidopsis thaliana *(TAIR 8.0; www.arabidopsis.org) and the closely related Euphorbiaceae *Ricinus communis *(TIGR; http://castorbean.jcvi.org/), as well as the non-redundant protein (NR) and nucleotide (NT) databases of GenBank ftp://ftp.ncbi.nlm.nih.gov/blast/db for those BLAST searches. To search for putative coding sequences and generate conceptual translations, we ran ESTScan with a pre-built model for *A. thaliana *that is distributed with the package. We also performed a Blast2GO analysis [5] to provide automatic annotation for unigenes using Gene Ontology terms according to BLASTX hits against GenBank NR database with a e-value threshold of 1e-10.

### Identification of gene expression patterns

To compare the expression of unisequences in developing and germinating seeds, the number of ESTs in each library was normalized by 10,000 reads. AC statistics [22] were used to estimate the significance of the differential expression.

### Annotation of lipid metabolism pathways

KAAS [14] and KOBAS [15] were used to annotate automatically *Jatropha *ESTs coding for orthologues to plant enzymes in the fatty acid biosynthesis, fatty acid degradation, triacylglycerol biosynthesis and triacylglycerol degradation pathways. The automatic annotations were enriched with searches for *Jatropha *orthologues to plant lipid metabolism enzymes annotated by PlantCYC [16] and the *Arabidopsis *Lipid Database [17] (TBLASTN/BLASTX bidirectional best hit; e-value 1e-5). All automatic annotations were visually inspected and edited as necessary.

## Competing interests

This research was supported by Petrobras SA, a Brazilian multinational energy company. The authors take complete responsibility for the integrity of the data and the analysis.

## Authors' contributions

GGLC performed the bioinformatics analyses, prepared the figures and drafted the final manuscript. KCC annotated the sequences and helped draft the manuscript. LEVDB prepared the first version of the manuscript, annotated the sequences and helped analyse the data. ACL annotated the sequences and helped draft the manuscript. MASC and FAC carried out the analyses related to the toxic components of the seed and helped draft the manuscript. LCL helped prepare the first version of the manuscript. FP critically revised and helped draft the manuscript. JAY and RV contributed to the bioinformatics analysis. RCM contributed to the metabolic pathway analyses. MJS conceived the study and carried out all of the experiments. All authors revised and approved the final manuscript.

## Supplementary Material

Additional file 1 **Automatic annotation of all unisequences using Blast2GO**. Click here for file

Additional file 2 **Correspondence between internal unisequence IDs and GenBank accession numbers**. Click here for file

Additional file 3 **Triacylglycerol biosynthesis pathway**. *Jatropha *enzymes found in the triacylglycerol biosynthesis pathway. The icons beside each enzyme name show the total number of *Jatropha *ESTs corresponding to that enzyme in the JD and JG, separately. See additional file 4 (oilpaths.pdf) for the unisequences assigned to each enzyme. Click here for file

Additional file 4 **Enzymes related to oil accumulation and breakdown in seeds**. Click here for file
