# Supplementary figures and images for: Transcriptome analysis of the oil-rich seed of the bioenergy crop Jatropha curcas L
Source: BMC Genomics. 2010 Aug 6;11:462. doi: 10.1186/1471-2164-11-462 (PMC3091658; doi:10.1186/1471-2164-11-462)

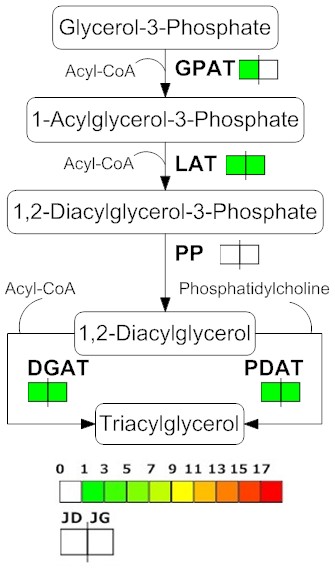

Supplement: Additional file 3 — Triacylglycerol biosynthesis pathway. Jatropha enzymes found in the triacylglycerol biosynthesis pathway. The icons beside each enzyme name show the total number of Jatropha ESTs corresponding to that enzyme in the JD and JG, separately. See additional file 4 (oilpaths.pdf) for the unisequences assigned to each enzyme. [file 1471-2164-11-462-S3.JPEG]
