# Supplementary material for: Transcriptome analysis of the oil-rich seed of the bioenergy crop Jatropha curcas L
Source: BMC Genomics. 2010 Aug 6;11:462. doi: 10.1186/1471-2164-11-462 (PMC3091658; doi:10.1186/1471-2164-11-462)
Supplement: Additional file 4 — Enzymes related to oil accumulation and breakdown in seeds. [file 1471-2164-11-462-S4.PDF]

Table S1 - Enzymes related to oil accumulation and breakdown in seeds

Number of unisequences assigned to enzymes of metabolic pathways related to oil accumulation and breakdown in the seeds.

Symbol	Enzymes	Unisequences	JD	JG	p-value[a]	NJD[b]	NJG[c]
		**Fatty Acid Biosynthesis**					
FatA	Acyl-ACP thioesterase A	Contig196	18	0	2.5e-05	24.59	0
		GJCCJC2057C09.b	1	0	6.1e-01	1.36	0
FatB	Acyl-ACP thioesterase B	GJCCJC2044C02.b	1	0	6.1e-01	1.36	0
ACC	Acetyl-CoA carboxylase	Contig655	5	0	5.7e-02	6.83	0
		GJCCJC2010F04.b	1	0	6.1e-01	1.36	0
		GJCCJC2027B01.b	1	0	6.1e-01	1.36	0
		GJCCJC2058F02.b	1	0	6.1e-01	1.36	0
EAR	Enoyl-ACP reductase	Contig200	2	0	3.4e-01	2.73	0
		JGCCJG2012A05.b	0	1	4.0e-01	0	1.68
HAD	Hydroxyacyl-ACP dehydrase	-	0	0	-	0	0
KAR	Ketoacyl-ACP reductase	Contig635	8	1	4.8e-02	10.92	1.68
		GJCCJC2004E05.b	1	0	6.1e-01	1.36	0
		GJCCJC2009D09.b	1	0	6.1e-01	1.36	0
		GJCCJC2011G11.b	1	0	6.1e-01	1.36	0
		GJCCJC2018H05.b	1	0	6.1e-01	1.36	0
KAS I	Ketoacyl-ACP synthase I	Contig644	3	0	1.9e-01	4.09	0
		Contig79	2	0	3.4e-01	2.73	0
		GJCCJC2054C12.b	1	0	6.1e-01	1.36	0
		GJCCJC2058B04.b	1	0	6.1e-01	1.36	0
KASII	Ketoacyl-ACP synthase II	Contig1291	3	1	5.2e-01	4.09	1.68
		GJCCJC2030D05.b	1	0	6.1e-01	1.36	0
KASIII	Ketoacyl-ACP synthase	-	0	0	-	0	0
MAT	Malonyl-CoA ACP transacyclase	GJCCJC2080C08.b	1	0	6.1e-01	1.36	0
FAD2	Oleoyl-ACP desaturase	Contig340	6	5	9.3e-01	8,19	8.43
		GJCCJC2013C09.b	1	0	6.1e-01	1.36	0
PCH	Palmitoyl-CoA hydrolase	JGCCJG2043B05.b1	0	1	4.0e-01	0	1,68
SAD	Stearoyl-ACP desaturase	GJCCJC2004A03.b	1	0	6.1e-01	1.36	0
		**Triacylglycerol Biosynthesis**					
LAT	1-Acylglycerol-3-phosphate-O acyltransferase	GJCCJC2046E05.b1	1	0	6.1e-01	1.36	0
DGAT	Acyl-CoA:diacyl-glycerol acyltransferase	GJCCJC2048D07.b1	1	0	6.1e-01	1.36	0
		JGCCJG2013H10.b	0	1	4.0e-01	0	1.68
GPAT	Glycerol-3-phosphate acyltransferase	GJCCJC2003H11.b	1	0	6.1e-01	1.36	0
PDAT	Phospholipid:diacyl-glycerol acyltransferase	JGCCJG2013H02.b	0	1	4.0e-01	0	1.68
PP	Phosphotidate phosphatase	-	0	0	-	0	0

OLE	Oleosin	Contig13	2	0	3.4e-01	2.73	0
		GJCCJC2072E05.b	1	0	6.1e-01	1.36	0
**Triacylglycerol Degradation**							
ML	Monoacylglycerol lipase	GJCCJC2013E10.b	1	0	6.1e-01	1.36	0
PLAS	Peroxysomal long-chain acyl-CoA synthetase	Contig1208	0	11	1.3e-04	0	18.55
		GJCCJC2010F01.b	1	0	6.1e-01	1.36	0
		GJCCJC2040B11.b	1	0	6.1e-01	1.36	0
		JGCCJG2007H01.b	0	1	4.0e-01	0	1.68
		JGCCJG2016E07.b	0	1	4.0e-01	0	1.68
PFAT	Peroxisomal fatty acid / acyl-CoA transporter	-	0	0	-	0	0
TL	Triacylglycerol lipase	Contig1070	0	6	7.2e-03	0	10.12
		Contig1303	0	12	5.8e-05	0	20.24
		Contig1544	0	2	1.8e-01	0	3.37
		Contig84	9	0	5.3e-03	12.29	0
		Contig867	2	0	3.4e-01	2,73	0
		GJCCJC2009F09.b	1	0	6.1e-01	1.36	0
		GJCCJC2018F09.b	1	0	6.1e-01	1.36	0
		GJCCJC2069F03.b	1	0	6.1e-01	1.36	0
		GJCCJC2078B02.b	1	0	6.1e-01	1.36	0
		GJCCJC2080E06.b	1	0	6.1e-01	1.36	0
		JGCCJG2011G08.b	0	1	4.0e-01	0	1.68
		JGCCJG2016F07.b	0	1	4.0e-01	0	1.68
		JGCCJG2018F11.b	0	1	4.0e-01	0	1.68
		JGCCJG2061A03.b	0	1	4.0e-01	0	1.68
**Fatty Acid Degradation**							
FADA	Acetyl-CoA acyltransferase	Contig1038	5	42	1.9e-10	6.83	70.84
		Contig1525	0	18	4.6e-07	0	30.36
		GJCCJC2004B10.b	1	0	6.1e-01	1.36	0
		JGCCJG2022G04.b	0	1	4.0e-01	0	1.68
		JGCCJG2025H04.b	0	1	4.0e-01	0	1.68
		JGCCJG2042D03.b1	0	1	4.0e-01	0	1.68
ATOB	Acetyl-CoA C-acetyltransferase	GJCCJC2008B09.b	1	0	6.1e-01	1.36	0
		JGCCJG2052C07.b	0	1	4.0e-01	0	1.68
ADH	Alcohol dehydrogenase	Contig1560	2	1	7.9e-01	2.73	1.68
ACAD	Acyl-CoA dehydrogenase	Contig1184	0	2	1.8e-01	0	3.37
		Contig1401	0	2	1.8e-01	0	3.37
ACOX	Acyl-CoA oxidase	Contig1167	0	2	1.8e-01	0	3.37
		Contig1600	0	2	1.8e-01	0	3.37
		Contig562	3	1	5.2e-01	4.09	1.68
		JGCCJG2002B12.b	0	1	4.0e-01	0	1.68
ALDH	Aldehyde dehydrogenase (NAD+)	Contig1080	0	4	3.6e-02	0	6.75
		Contig1337	0	2	1.8e-01	0	3.37
		Contig6	6	2	3.0e-01	8.19	3.37
		JGCCJG2039G05.b1	0	1	4.0e-01	0	1.68
DCR	Dienoyl-CoA reductase	Contig1191	0	9	6.4e-04	0	15.18
		GJCCJC2021C11.b	1	0	6.1e-01	1.36	0
		JGCCJG2063E05.b	0	1	4.0e-01	0	1.68
		JGCCJG2010C10.b	0	1	4.0e-01	0	1.68

		GJCCJC2005H04.b	1	0	6.1e-01	1.36	0
PAAG	Enoyl-CoA hydratase	Contig1260	0	4	3.6e-02	0	6.75
		Contig1523	0	3	8.0e-02	0	5.06
		Contig482	3	14	1.9e-03	4.09	23.61
		Contig1145	0	2	1.8e-01	0	3.37
		Contig1410	0	2	1.8e-01	0	3.37
		GJCCJC2042A10.b	1	0	6.1e-01	1.36	0
		GJCCJC2016E01.b	1	0	6.1e-01	1.36	0
		JGCCJG2028A05.b	0	1	4.0e-01	0	1.68
		GJCCJC2013D11.b	1	0	6.1e-01	1.36	0
		JGCCJG2034A11.b	0	1	4.0e-01	0	1.68
		GJCCJC2080F12.b	1	0	6.1e-01	1.36	0
		JGCCJG2063H12.b	0	1	4.0e-01	0	1.68
ACSL	Long-chain acyl-CoA synthetase	GJCCJC2010F01.b	1	0	6.1e-01	1.36	0
		GJCCJC2050G11.b	1	0	6.1e-01	1.36	0
		JGCCJG2007H01.b	0	1	4.0e-01	0	1.68
		JGCCJG2016E07.b	0	1	4.0e-01	0	1.68

[a] The p-value refers to the Audic-Claverie statistics for differential expression
[b] JD normalised to 10,000 reads
[c] JG normalised to 10,000 reads
